# Low Gluten Beers Contain Variable Gluten and Immunogenic Epitope Content

**DOI:** 10.3390/foods12173252

**Published:** 2023-08-29

**Authors:** Mitchell G. Nye-Wood, Keren Byrne, Sally Stockwell, Angéla Juhász, Utpal Bose, Michelle L. Colgrave

**Affiliations:** 1 School of Science, Edith Cowan University, Perth, WA 6027, Australia; m.nyewood@ecu.edu.au (M.G.N.-W.); a.juhasz@ecu.edu.au (A.J.); utpal.bose@csiro.au (U.B.); 2 Australian Research Council Centre of Excellence for Innovations in Peptide and Protein Science, Perth, WA 6027, Australia; 3 CSIRO Agriculture and Food, St. Lucia, QLD 4067, Australia; keren.byrne@csiro.au (K.B.); sally.stockwell@csiro.au (S.S.)

**Keywords:** beer, coeliac disease, contamination, ELISA, gliadin, gluten, hordein, liquid chromatography, mass spectrometry, proteomics

## Abstract

Gluten content labels inform food choice and people practicing a gluten-free diet rely upon them to avoid illness. The regulations differ between jurisdictions, especially concerning fermented foodstuffs such as beer. Gluten abundance is typically measured using ELISAs, which have come into question when testing fermented or hydrolysed foodstuffs such as beer. Mass spectrometry can be used to directly identify gluten peptides and reveal false negatives recorded by ELISA. In this survey of gluten in control and gluten-free beers, gluten protein fragments that contain known immunogenic epitopes were detected using liquid chromatography-mass spectrometry in multiple beers that claim to be gluten-free and have sufficiently low gluten content, as measured by ELISA, to qualify as being gluten-free in some jurisdictions. In fact, several purportedly gluten-free beers showed equivalent or higher hordein content than some of the untreated, control beers. The shortcomings of ELISAs for beer gluten testing are summarised, the mismatch between ELISA and mass spectrometry results are explored, and the suitability of existing regulations as they pertain to the gluten content in fermented foods in different jurisdictions are discussed.

## 1. Introduction

Gluten proteins consist of prolamins found in wheat, barley, rye, and oats [1]. These proteins contain specific immunogenic epitopes [2] that trigger an inappropriate immune reaction in the intestine of people with coeliac disease (CD). In those with CD, gluten proteins are incompletely digested and modified by tissue transglutaminase in the small intestine, which enhances the stimulation and recruitment of proinflammatory T cells in the lamina propria [3]. This, in turn, leads to mucosal injury and causes a range of intestinal symptoms ranging from mild digestive issues to more severe complications such as malnutrition and anaemia [4]. Epitopes that are immunogenic can vary between individuals with CD, though there are known core epitopes that affect 97.5% of patients or higher [2]. In CD patients, as little as 50 mg gluten intake per day damages the intestinal mucosa [5], and to avoid this, a strict gluten free diet must be followed. The ability to do this is, in part, dependent upon the accuracy of gluten content labelling in food, which varies between jurisdictions. There are concerns that beer deemed as ‘low gluten’ (20–200 mg/kg gluten) or gluten free (<20 mg/kg) can elicit symptoms in people with CD [6]. 

Food safety labelling regulations vary across jurisdictions, though the Codex Alimentarius represents a consensus of regulatory standards [7]. It recommends all foodstuffs containing 20–100 mg/kg (or ppm) gluten be labelled as ‘very low gluten’ to reflect their relative safety for those with mild CD or related intolerances, while the label ‘gluten free’ be reserved for those with less than 20 mg/kg gluten. The Gluten Free Certification Organization, a third-party certification program administered by the Gluten Intolerance Group, practises a stricter 10 mg/kg threshold for gluten-free accreditation [8]. The 20 mg/kg threshold is reflected in legislation in Europe, the UK, and the USA [9], but is less restrictive than Food Standards Australia and New Zealand (FSANZ) Code, Schedule 4 (1991, r. 2017), which defines “gluten free” as a nutrition content claim where the product must have no detectable gluten. FSANZ also requires the foodstuff to carry no risk of gluten cross contamination, and no oats, wheat, barley, rye, or their products as ingredients. Foods labelled “low gluten” must have less than 200 mg/kg gluten. 

The sandwich enzyme-linked immunosorbent assay (ELISA) is the official method for gluten determination for both the Codex Alimentarius and AOAC International (US). This requires the target antigen to have two or more antibody-binding epitopes per molecule. However, in fermented or heated food products wherein gluten proteins may be digested or fragmented to peptides and thus may only contain a single epitope per molecule, a competitive ELISA is more appropriate [10]. There are five antibodies used in various ELISA kits to detect gluten proteins [11,12,13], two of which have been internationally ring-tested [14], namely, the R5 antibody raised against rye prolamins [15,16,17] and the Skerritt antibody (monoclonal antibody (mAb) 40,121) raised against omega-gliadins [18,19]. Only a single type, the R5 antibody, is listed in the Codex Alimentarius as being suitable for gluten determination. These monoclonal antibodies react to specific peptides, which occur in different frequencies in wheat, barley, and rye protein fractions and lead to different gluten estimates to a standard sample [20,21,22,23]. Further differences in measured gluten content arise from gluten extraction procedures, standard materials, and the design of different ELISA kits [10,24,25,26,27]. 

Notably, the R5 antibody does not accurately quantify barley prolamins or hydrolysed gluten [28]. While competitive ELISAs theoretically detect single epitopes on hydrolysed peptides [29], the competitive assay using the R5 antibody requires polyphenol containing samples such as beer to have protein extracted in an ethanol solution that is unable to extract all types of prolamins when they have been denatured [28]. Furthermore, gelatine is recommended in the extraction solution to avoid non-specific interactions between polyphenols and antibody. Denatured prolamins in finished beer may therefore be absent or under-represented in the tested extract, leading to artificially low readings and/or false negatives. 

Beer production involves multiple steps where prolamins can be digested, degraded, or sequestered, including malting, mashing, filtering, fermenting, and stabilisation [30]. Remaining gluten concentrations are typically above CD regulatory thresholds [31], but can be decreased by using highly modified malt [32] or by using flocculating agents such as polyvinylpolypyrrolidone [30,33], silica gel, or tannins [34]. Alternatively, enzymes can be added [35] such as prolyl endopeptidases (PEPs) to digest proline-rich gluten proteins directly [36,37], or transglutaminase, which crosslinks dissolved proteins and facilitates their precipitation and removal [34,38]. Gluten can be virtually eliminated by using novel custom-bred ultra-low gluten (5 mg/kg) barley varieties [39] to make beer [14], and truly gluten-free fermented drinks that resemble beer can be made using pseudocereals or adjunct carbohydrate sources, although co-mingling of grains during crop rotation or food production can render otherwise gluten-free cereals a risk for CD patients [40]. Despite orders of magnitude decreases in beer gluten protein content being possible with these methods, related proteins are still detectable using ELISA or liquid chromatography-mass spectrometry (LC-MS). These techniques offer complementarities as well as unique advantages and challenges [41], and are both utilized in the current study. 

The technicalities involved in beer production, and then measuring and regulating gluten protein content in beer is a topic of research. The USA’s FDA has recently issued a ruling on the gluten-free labelling of fermented foods such as beer that recognises this difficulty [42]. They ruled that for partially digested or modified foodstuffs such as beer to be denoted as gluten-free, their ingredients must be gluten-free prior to fermentation, and there must not be any possibility of cross-contamination during production. The gluten status of raw ingredients is tested using established techniques, the risk of accidental gluten contamination during production is controlled to some degree through regulated production practices, and inadvertent contamination during production is tested for by measuring the finished foodstuff. This approach is practical in its application of gluten testing and relies on administrative controls when necessary. However, it may be overly conservative when beers are designed to have gluten removed, and it may fail to detect contaminating gluten proteins that are partially digested or denatured and are therefore under-represented by currently available ELISAs [28]. There is, therefore, a need to survey the beer proteome for gluten content using LC-MS to inform the scientific community, regulators, and the public of the efficacy of existing regulations and production practices. The present study assesses the gluten content of specialised low-gluten beers made using different ingredients and/or production techniques and aims to substantiate consumer trust in products and regulations, while identifying gaps in current gluten detection techniques. 

## 2. Materials and Methods

### 2.1. Materials 

Reagents were sourced from Sigma-Aldrich (Sydney, NSW, Australia), solvents from ChemSupply (Gillman, SA, Australia), and enzymes used for digestion (trypsin and chymotrypsin) from Promega (Sydney, NSW, Australia). The Ridascreen R5 competitive ELISA kits were purchased from R-Biopharm Australia (Caringbah, NSW, Australia) and were performed according to manufacturers’ instructions. 

### 2.2. Sample Collection 

A range of beers (single bottles) were selected that included different types of malt and brewing styles, as well as regular (control), low, and reduced-gluten labelling. They were chosen based on their advertised ingredients (barley malt) and gluten status claims and purchased from international commercial vendors in 2020. Two of the gluten-reduced beers were selected based on anecdotal evidence by consumers of symptoms induced by consumption. Five regular beers that had been previously confirmed to have a range of gluten content [14] were used as positive controls, C1–C5. Nine beers were advertised as gluten free or low gluten (LG), LG1–LG9. LG1 is a German pilsner that uses gravitation, cold, and filtration to lower gluten content. LG2 is an Australian low-carb, low-gluten pale lager. LG3 and LG4 are British beers that use silica technology to remove gluten. LG5 and LG6 are Finnish beers that claim a filtration process. LG7, LG8, and LG9 are Finnish beers that use an undisclosed method to achieve gluten-free labelling status. 

### 2.3. In-Solution Enzymatic Digest 

To digest whole beers (*n* = 4 technical replicates) using either trypsin or chymotrypsin, aliquots of degassed beer (50 μL) were diluted in 50 μL of 50 mM ammonium bicarbonate containing 1 mM CaCl_2_, pH 8.5. To the beer, 10 μL of 50 mM dithiothreitol was then added, and the samples were incubated for 30 min at 60 °C. The samples were cooled, 10 μL of 100 mM iodoacetamide was added, and the samples were then incubated at 20 °C for 20 min in the dark. To each solution, 10 μL of either trypsin or chymotrypsin (1 μg/μL) was then added and the samples were incubated at 37 °C for 16 hours. To quench the digestion, 50 μL of 1% formic acid (FA) was added, and the samples were frozen at −20 °C until analysis. 

### 2.4. Discovery Proteomics

The samples were analysed on a 6600 TripleTOF MS (SCIEX, Redwood City, CA, United States) coupled to an Ekspert nanoLC415 (Eksigent, Dublin, CA, USA). Aliquots measuring 5 μL were loaded onto a ChromXP C18 trap column (3 μm, 120 Å, 10 × 0.3 mm), using 10 μL/min 0.1% FA for 5 min. Peptides were chromatographically separated on a ChromXP C18 (3 μm, 120 Å, 150 × 0.3 mm) column at a flow rate of 5 μL/min. Mobile phases A (5% DMSO, 0.1% FA, 94.9% water) and B (5% DMSO, 0.1% FA, 90% acetonitrile, 4.9% water) varied in a linear gradient from 5% to 45% solvent B over 40 min, then 45–90% B over 5 min, a 5 min hold at 90% B, return to 5% B over 1 min, and 14 min of re-equilibration. The HPLC eluent was directed onto a DuoSpray ion source with heated interface set to 100 °C, ion spray voltage to 5500 V, curtain gas to 20 psi, and ion source gas 1 and 2 set to 15 and 20 psi, respectively. 

Data were acquired in information-dependent acquisition mode, where a time-of-flight MS spectrum was recorded over mass range m/z 350–1800 with 0.25 s accumulation time. The 30 most intense precursor ions that exceeded 200 counts per second, with charge state between 2–5, were selected for tandem mass spectrometry in 30 tandem MS scans (mass range m/z 100–2000, 0.04 s accumulation time each). Dynamic ion exclusion was used to avoid selecting a precursor ion after one occurrence within 8 s, with ions having a mass tolerance 50 ppm and peaks within 6 Da of the precursor being excluded. 

Raw data were searched using ProteinPilot software v5.0.3 (SCIEX) against a database consisting of all Poaceae entries available on UniProtKB (accessed on 21 February 2023), concatenated with common repository of adventitious proteins (thegpm.org/crap). This identified tryptic and chymotryptic peptides detected in the samples. Search results are reported at 1% global false discovery rate as determined by an algorithm incorporated into ProteinPilot [43].

### 2.5. Targeted Proteomics 

Samples (10 μL) were injected onto a UHPLC system (Shimadzu Nexera, Sydney, Australia) directly coupled to a QTRAP 6500 mass spectrometer (SCIEX) and peptides from barley hordeins and wheat glutenins and gliadins were targeted using multiple reaction monitoring (MRM) analyses to examine the LG beers and compare gluten content to control beers (C1-C5). The MRM assay cycle time was 0.3 s, and transitions were scheduled to be ± 40 s of their observed retention time. 

MRM data were analysed using Skyline v.21.2.0.425 [44], where extracted ion chromatograms were generated for each transition and peak areas exported to Microsoft Excel. Transition lists for barley-specific hordeins and, separately, wheat-specific gluten peptides known to ionise well at low abundance [45] were assessed. Accessions were derived from UniProtKB and an additional tryptic peptide from γ-hordein TC131355 and a tryptic and a chymotryptic peptide from B-hordein TC138764 were monitored using translated protein sequences originally published in the TIGR database of repetitive sequences in plants [46]. Three transitions were monitored per peptide, and their peak areas were summed to derive relative abundance of peptides. Variability was assessed by calculating the coefficient of variation for each peptide across the four technical replicates. Where peptides had a Met residue, which is reduced during sample preparation but may become oxidised, precursor ions with both oxidised (+16 Da) and reduced Met were targeted. 

### 2.6. Statistical Analysis and Visualisation

Peptide peak area was exported from Skyline into Excel for targeted tryptic and chymotryptic assays. The mean peak area and standard deviation (SD) of each protein class (γ-, B-, C-, D-hordein, or ALPs) was determined across the four technical replicates for each beer and graphs were generated in GraphPad Prism (mean value ± SD). Heatmaps of peptide abundance were visualised and annotated using the web-based software Morpheus (https://software.broadinstitute.org/morpheus/). Wheat peptide MRM data was similarly exported from Skyline to GraphPad where mean ± SD were plotted for each beer directly. 

### 2.7. Epitope Mapping

HLA-DQ core and other allergenic epitope sequences in barley and wheat from Sollid et al. (2012), updated 2020 [47,48], were collected into a motif list in CLC Main Workbench (Qiagen, Aarhus, Denmark). The R5 epitopes (QQPFP, QQQFP, QLPFP, LQPFP [16]) were added to detect epitopes that would be expected to elicit an ELISA signal. Additional T cell epitopes identified by Tye-Din et al. (2010) were also added [49]. To reveal those epitopes that may be present in the identified proteins of the beer samples, the epitope motif list was searched against FASTA files of the proteins detected in each beer according to the tryptic and chymotryptic discovery data. To limit the search for epitopes to those peptides that were directly detected, another FASTA file of tryptic and chymotryptic peptides detected with 99 conf was generated for each LG beer and searched with the epitope motif list. This provided a conservative assessment of allergenic epitopes detected in each LG beer. For reference purposes, the barley reference proteome (Hordeum vulgare Morex v3, UniProtKB UP000011116) was accessed and searched with the epitope motif list. 

## 3. Results

### 3.1. Measuring Gluten Protein Content in Beer Using Competitive ELISA

Degassed beers were measured using an R5 competitive ELISA (Ridascreen, R-Biopharm AG, Germany) according to the manufacturers’ instructions. As listed in Table 1, all LG beers recorded values below the Codex threshold of 20 mg/kg gluten. Of the five control beers, C4 and C5 also met this gluten-free threshold while C1 and C3 returned between 20 and 100 mg/kg gluten which fulfills the Codex criteria to be labelled ‘gluten-reduced’, ‘very low gluten’, or similar according to national regulations. Only one beer, C2, was above all thresholds, at 146 mg/kg. 

### 3.2. Discovery Proteomics on Trypsin- and Chymotrypsin-Digested Beer Samples

LG beers were subjected to discovery proteomics using data-dependent LC-MS acquisition, which detected tryptic and chymotryptic peptides separately. The high sequence homology of gluten proteins means that different proteins include the same peptides in their sequence, and multiple proteins may be present in each protein group in bottom-up proteomics [50]. The majority of the detected peptides and proteins originated from non-gluten protein types such as serpins or lipid transfer proteins, others were from avenin-like proteins which also belong to the prolamin protein family, and several were from hordeins. Most of the detected peptides were from barley or shared between barley and other Poaceae species; however, a small number of wheat-specific peptides were detected, including, but not limited to, chymotryptic RIEMPGPPY plus the related ACRIEMPGPPY and VEEQACRIEMPGPPY from a CM17 protein (Q41540), tryptic DAEGQLPSRT and PDEKDAEGQLPSR from a peroxiredoxin (Q6W8Q2), and peptide VEVEDGNILQISGERK from a small heat shock protein domain-containing protein (A0A3B6JKI7). Many peptides common to several Poaceae species were detected (Tables S1 and S2). This suggests that wheat may have been inadvertently incorporated as a raw ingredient or as a contaminant in the production process. 

There were 2091 distinct peptides detected across all LG beer tryptic digests, which identified at least 199 protein groups (excluding cRAP proteins). The top identified proteins include serpins, lipid transfer proteins (LTPs), and alpha-amylase/trypsin inhibitors, as well as B-, D- and γ-hordeins (Table S1). 

In chymotryptic digests of LG beers, there were 787 distinct peptides and 110 protein groups detected. Serpins, D-, B-, and γ-hordeins, LTPs, amylase inhibitors, and trypsin inhibitors were seen in the top 10 proteins, as is typical of beer [14] (Table S2). 

#### 3.2.1. Identification of Hordein Proteins in LG Beer 

**Figure 1:**
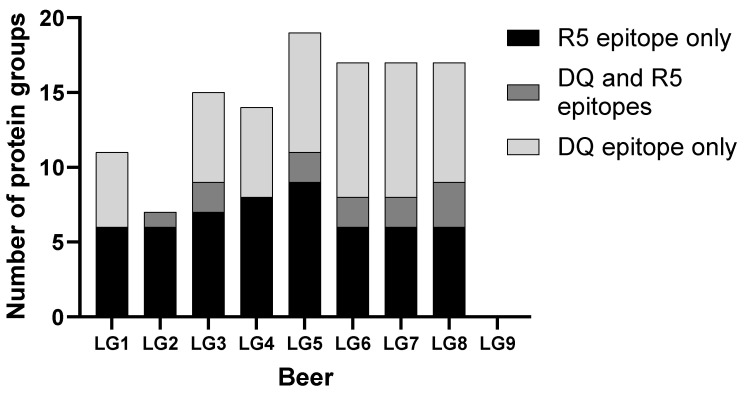
The number of proteins detected in each LG beer with DQ core and/or R5 epitopes mapping to them. This reveals the small degree of overlap between R5 and DQ epitopes on the proteins detected in LG beers. All LG beers had between 6 and 9 proteins that contain R5 epitopes, except for LG9.

To determine how many potentially immunogenic proteins are detectable in the LG beers, the proteins detected in each beer were searched for known immunogenic epitopes. The protein sequences in each protein group were searched for R5 epitopes [16], HLA-DQ core and additional coeliac disease-specific HLA-DQ epitopes [47,48], and gluten protein T cell epitopes with known strength of immune response [2,49]. Because the peptides used to detect the proteins are present in multiple Poaceae proteins, some but not all of which are necessarily present in the LG beers, proteins were grouped according to the Paragon algorithm in ProteinPilot to give a conservative estimate of the number of immunogenic proteins that remain detectable in LG beers. Most R5-, DQ core-, and HLA-DQ epitope-positive protein groups were detected in both the tryptic and chymotryptic digests but were counted only once to determine the overlap between the number of proteins containing HLA-DQ and/or R5 epitopes (Figure 1).

Proteins with immunogenic epitopes were detected in all LG beers except for LG9 (Figure 1). While the R5 epitopes recognised by the R5 ELISA were also detected in these beers, they usually occurred in different proteins to those containing immunogenic epitopes. The R5 ELISA will therefore produce a signal in response to barley proteins but may not detect immunogenic proteins directly. For comparison, in the barley Morex v3. proteome containing 35,907 proteins, 48 instances of R5 epitopes appear on 28 proteins. 

#### 3.2.2. Identification of Hordein Peptides in LG Beer

**Figure 2:**
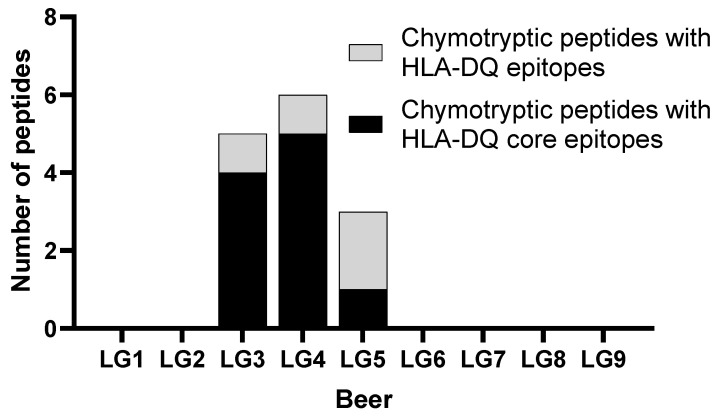
The number of distinct peptides detected in chymotryptic samples that contain intact DQ core immunogenic epitopes [48], and additional matches to linear epitopes with confirmed, positive assays filtered for the HLA-DQ alleles characteristic of CD [51] that are nine amino acids or longer. The immunogenic peptides occur on multiple proteins but counted once. Tryptic samples had zero matches to Poaceae antigens. These peptides contain immunogenic epitopes that are not cleaved by trypsin or chymotrypsin, which are incorporated into sample preparation and are also natural intestinal proteases.

Immunogenic epitopes (e.g., HLA-DQ and R5) in the intact protein may be destroyed in the tested sample, either from hydrolysis during the beer production process (by endogenous enzymes during malting, by exogenous enzymes such as AN-PEP added during fermentation), or intentionally during sample preparation. However, both enzymes used in sample preparation (trypsin and chymotrypsin) are present in the human digestive system, so any beer proteins consumed would be expected to be cleaved by the same enzymes in vivo. Peptides that are cleaved but retain 9-amino acid core epitope regions will exhibit immunogenic properties and are potentially clinically relevant [48]. Figure 2 presents the numbers of peptides detected that contain intact DQ core epitopes [48], and confirmed immunogenic epitopes (matching at least nine residues of the epitope region) [51] in discovery proteomics data. Tryptic samples had zero matches to Poaceae antigens.

### 3.3. Targeted MRM-MS for Hordein Quantification

**Figure 3:**
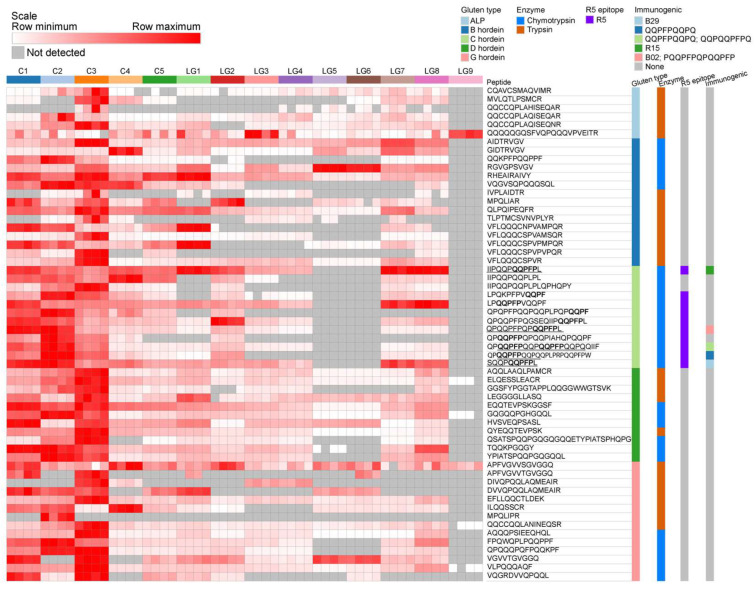
Relative abundance of targeted peptides in control (C) and low gluten (LG) beers, annotated with peptide sequence, gluten protein type, the dataset in which it was measured (trypsin or chymotrypsin), R5 epitope status, and immunogenic epitope status. Intact R5 epitopes are written in bold text. Immunogenic epitopes are underlined, and match epitopes B02, B12, and R15 from Tye-Din et al. (2010) [49], three listed in Sollid et al. (2012) [47], and PQQPFPQPQQPFP, which matched IEDB epitope ID 226653. Cell colors where the peptide was not detected are colored in grey, and others are colored using a white-to-red scale. G-hordeins refer to γ-hordeins.

MRM assays were used to quantify gluten-derived peptides from hordeins and avenin-like proteins (ALPs) in trypsin- and chymotrypsin-digested samples that yielded high responses. MRM allows for quantitative and more sensitive measurements of peptides than DDA-MS. Figure 3 presents abundance values of peptides that were not reported by discovery proteomics (Figure 1 and Figure 2). 

Of the peptides monitored in Figure 3, many that belong to the same gluten type show patterns of presence and absence that may be attributable to the barley variety used in the beer, or to the manufacturing process. For example, the γ-hordein peptide ILQQSSCR is very low in C3 but abundant in C4, unlike most other γ-hordein peptides. Such a result may instead arise from the brewing process if complete hydrolysis or removal is achieved in some, but not all, beers. 

ALP protein levels were generally higher in control than in LG beers and were seen in all nine LG beers. Peptide QQQQQGQSFVQPQQQVPVEITR was seen in LG3 and LG9 at higher levels than in control beers, and was the only ALP detected in Finnish beer LG9. 

Members of the B-hordein family share high sequence homology, meaning that their peptides (both tryptic and chymotryptic) are markers of multiple B-hordein isoforms. While overall B-hordein content is more abundant in control than in LG beers, selected peptides were more abundant in LG beers than in control (Figure 3). Two peptides, VFLQQQCSPVPMPQR and RHEAIRAIVY, are highest in LG1, and RGVGPSVGV is highest in LG5, followed by LG6. Notably, peptides RHEAIRAIVY and QQKPFPQQPPF are present in P06470, a B1-hordein, and while the former is detected in LG3, LG4, LG5, and LG6, the latter is not, suggesting that this part of the protein is digested in some beers. Despite targeted B-hordeins being abundant in most beers, none were detected in LG9. 

C-hordeins have over 20 isoforms per genome [52], but are predominantly detected using the chymotrypsin MRM assay because their sequences are largely repetitive and are poor in trypsin cleavage sites. Twelve chymotryptic C-hordein peptides were targeted in the analyses (Figure 3). Three (IIPQQPQQPFPL, QPQQPFPQPQQPFPL, and SQQPQQPFPL) are partial matches to rye peptide R15, barley peptide B02, and barley peptide B29, respectively, that elicit symptoms in CD patients [49]. One of these and two more peptides contain DQ core immunogenic epitopes. Immunogenic peptide IIPQQPQQPFPL is more abundant in LG7 and LG8 than in all control beers, and is present in LG1, LG2, LG3, and LG4. Intact R5 epitopes are present on these five immunogenic peptides and several other monitored C-hordein peptides. R5 epitope-containing C-hordein peptides are present in all beers except LG5, LG6, and LG9. C-hordein peptides are notably absent in LG5, LG6, and LG9. 

D-hordein presents as a single gene in barley [53,54], encoding proteins rich in Pro and Gln residues that are orthologous to x-type and y-type wheat high molecular weight (HMW) glutenins. All tryptic and all but one chymotryptic peptides that were monitored were detected in all control beers (C1-C5) and were seen at varying levels in LG beers. LG9 had notably less D-hordein peptide content, with only two peptides detected at low levels, tryptic ELQESSLEACR and chymotryptic GQGQQPGHGQQL. Two C-terminal tryptic peptides, AQQLAAQLPAMCR and LEGGGGLLASQ, and two N-terminal peptides, ELQESSLEACR and HVSVEQPSASL, were detected broadly (in all LG beers except LG9), while more repetitive region-originated peptides were detected at relatively low levels in some, but not all, LG beers.

Barley γ-hordeins have three isoforms (γ-1, -2, and -3) and are potential triggers of CD, but are less abundant than the B- and C-hordeins [55]. Some variation in the peptides detected are likely due to genetic variability of varieties used in brewing, as none of the control beers contain all 14 monitored peptides. Similarly, γ1-hordein peptide APFVGVVTGVGGQ was detected in only four beers while APFVGVVSGVGGQ, which differs at a single residue, was present in all beers, including LG9. The latter of these two peptides was previously identified from the TIGR Plant Repeat Database and is unique to the γ1-hordein with NCBI reference sequence number XP_044947070.1 and UniProt accession A0A8I6WAD5. 

### 3.4. Measurement of Relative Abundance of Gluten Proteins

**Figure 4:**
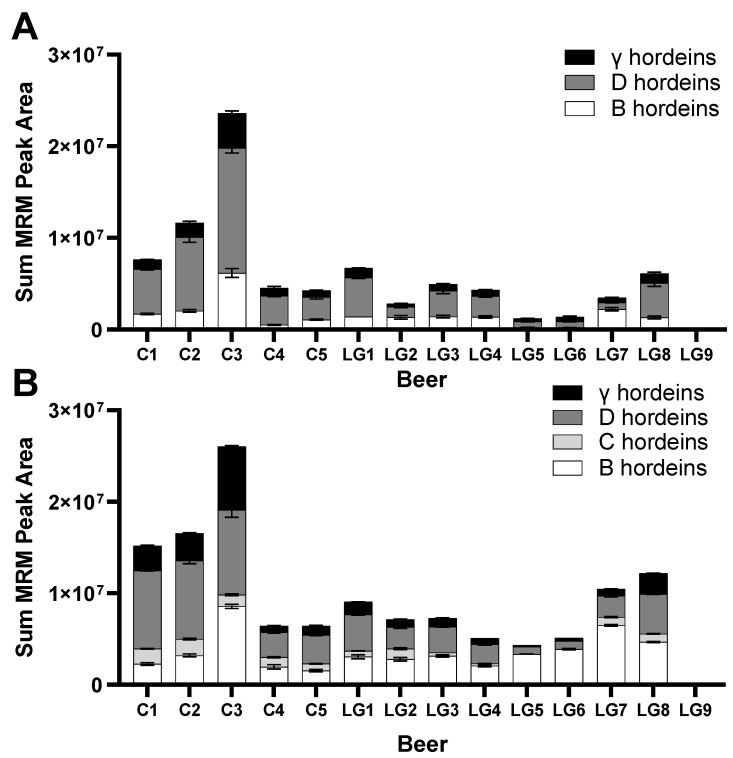
Net peak area of monitored hordein and ALP peptides from the (**A**) tryptic and (**B**) chymotryptic digested samples across control beers (C1–C4) and low gluten (LG) beers tested. The peak area is an indication of abundance but is not a direct measure of gluten content.

The relative abundances (as measured by the peptide peak area) of the gluten protein peptides in Figure 3 can be summed to estimate each beer’s overall relative hordein content. While the list of monitored peptides is not absolute, and other hordein peptides likely exist in the beers, the set that was monitored in Figure 3 represents peptides that ionise well and can serve as sensitive hordein subtype-specific markers that reflect the most-abundant hordein peptides as measured by LC-MS. Summing all monitored peptide peak areas allows for a comparison of the relative gluten protein content independent of the different barley varieties being used as ingredients in these beers. This was performed for both tryptic (Figure 4A) and chymotryptic (Figure 4B) B-, C-, D-, and γ-hordein specific peptides. ALPs were excluded because they are not strictly hordeins, and ALPs identified in the barley Morex v3 proteome lack R5 epitopes. 

The differences in net hordein protein peak area between beers is evident in both tryptic and chymotryptic digests (Figure 4). In most cases, D-hordein accounts for most of a beers’ hordein peptide signal (peak area). C3 is the beer with the highest hordein peptide signal, followed by C2, then C1, as expected for untreated control beers. Strikingly, LG8, LG1, and LG7 have the fourth, fifth, and sixth highest hordein net peak areas, respectively, and have more than control beers C4 and C5. Beers LG2, LG3, and LG4 contain a similar level to C4 and C5, while LG beers LG6 and LG5 are lower. Levels in LG9 are the lowest, orders of magnitude below the others. Comparing the trypsin and chymotrypsin results shows similar patterns of relative abundance. It should be noted that the peptides used are different and their resultant responses (MS peak area) are expected to vary according to the peptide sequence, which directly impacts the ionisation potential. 

### 3.5. Wheat Detection

**Figure 5:**
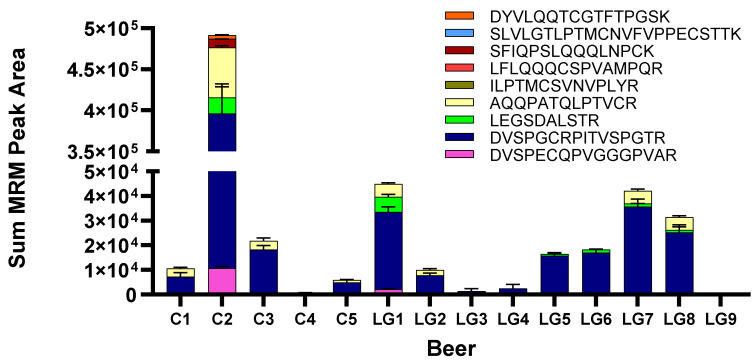
Tryptic wheat-specific peptides detected in control (C) and LG beers. C2 had an order of magnitude higher wheat content and is shown on a split axis. A single peptide, DVSPGCRPITVSPGTR, was seen in C4, LG3, and LG4 and none were detected in LG9. Multiple peptides were seen at low levels in all other beers.

Since wheat-specific proteins were observed in most LG beers during discovery proteomics, the beers were subjected to MRM analysis targeting nine wheat-specific peptides that ionise well to test for potential contamination [56]. Wheat-specific HMW glutenin peptides were detected in all LG beers except for LG9 (Figure 5). In beer C2, all nine monitored peptides were consistently observed as expected with wheat as an ingredient. In C4, LG3, and LG4, low levels of a single peptide (DVSPGCRPITVSPGTR) were detected, and in LG9, no peptides were detected. In other beers (C1, C3, C5, LG1, LG2, LG5, LG6, LG7, LG8), multiple peptides were detected at relatively low abundance. The detection of wheat proteins in LG beers implies contamination of the production line with wheat and the incomplete digestion or removal by filtration or other means during beer production.

## 4. Discussion

Gluten proteins were detected and measured in nine LG and five control beers by ELISA and targeted LC-MS. While the relative gluten protein abundance varies greatly between beers, there were differences within control and LG groups, between the types of hordeins monitored, and between the measurement method (Figure 3, Table 1). This mismatch was observed between the ELISA result, which estimated gluten content to be less than 20 mg/kg in all LG beers and some control beers, and LC-MS results, where several LG beers contained net hordein content comparable to high-gluten control beers. Notably, several intact immunogenic epitopes were seen in LG beers at levels comparable to control beers (Figure 2 and Figure 3), and five LG beers contained multiple peptides specific to the wheat proteome, indicating contamination had occurred and highlighting a unique risk for CD food safety regulation.

Gluten-free labelling regulations are traditionally based on antibody ELISA measurements, such as those shown for the competitive R5 ELISA assay results presented in Table 1. The R5 antibody recognises sequences such as QQPFP, QQQFP, LQPFP, and QLPFP [16]. Despite these peptides being detected intact in several C-hordein peptides, and both chymotrypsin and trypsin data indicating that peptide LQPFP was also found in the beta-amylase (AMYB) present in both LG5 and LG6 beers, all LG beers were below 20 mg/kg gluten by ELISA measurement, qualifying as ‘gluten-free’ according to the Codex Alimentarius [7]. Two of the five control beers, C4 and C5, also met the gluten-free criteria, and the other two control beers, C1 and C3, were between 20–100 mg/kg, so they can be labelled as ‘gluten reduced’ or similar, according to jurisdiction. By itself, these ELISA results showing low gluten protein content might be interpreted as evidence that some commercial brewing recipes are effective at lowering gluten content similar to specialised methods, however, given that the relative hordein abundance between the 14 beers is different when measured by LC-MS (Figure 4), there may be a confounding variable. 

Epitope masking by phenolic compounds can hinder the interaction between anti-bodies and epitopes. Polyphenols extracted from cranberry or apple inhibit signal from anti-gliadin antibodies in dot blot assays, demonstrating a reversible and dose-dependent interaction between certain polyphenols and gliadins mediated by epitope masking and potentially insoluble protein-polyphenol complexes [57]. Similar observations have been made using phenolic compounds to lower IgE-mediated responses in peanut allergies [58,59]. While insoluble protein-polyphenol complexes cause haze in beer [60] and can be removed by cold filtration, a similar dose-dependent epitope-masking phenomenon has been demonstrated in beer arising from native beer components [41]. Beer filtrates containing proteins between 30–100 kDa, as well as a smaller <3 kDa fraction suppressed signals from sandwich ELISA. The 30–100 kDa fraction was thought to be enriched in hordein oligomers or glycated monomers. The <3 kDa fraction was thought to include hordein fragments; however, given that results were similar for three beers with different levels of hordein fragments detected using LC-MS, polyphenols less than 3 kDa in size cannot be ruled out as causative agents [41]. Phenolic phytochemicals are known to interact promiscuously and non-specifically with proteins [61], and may contribute to epitope masking in vitro. This would imply that attempts to lower beer gluten content by digesting hordeins may be prone to false negatives, due to suppression of ELISA signals by hordein fragments or polyphenols. To address this, the manufacturer’s instructions for the R5 ELISA assay recommends adding foreign proteins such as skim milk powder [62] or fish gelatine [63] during extraction or testing to suppress matrix effects when analysing polyphenol-containing substrates such as beer. Further research into the ability of polyphenols in beer to suppress ELISA signals is warranted. 

There is evidence for low levels of wheat content based on four wheat peptides from three wheat HMW glutenin isoforms (Q45R38, Q6RX92, and Q41553) in six of the LG beers: LG1, LG2, LG5, LG6, LG7, and LG8 (Figure 5). Notably, six wheat peptides were detected in beer C2. The source of this wheat detection is as a known ingredient. The detection of a trace amount of wheat may occur because of agricultural co-mingling (in field, storage, or during transport), contamination during processing, or from other ingredients such as yeast. For the trace levels of wheat proteins seen in LG1, LG2, LG5, LG6, LG7, and LG8, the source is unclear. If the yeast was cultured on wheat substrate, as is common [64], and was added immediately prior to bottling, it is a possible source of wheat proteins. While a low-gluten brewery could source yeast not propagated using wheat, this contamination may occur elsewhere in the production process, such as the hops supply, the malthouse, or the barley field. 

C-hordein peptides were detected in most of the LG beers, except for LG5, LG6, and LG9. The Finnish beers, LG7 and LG8, showed elevated levels of C- hordeins, and LG2 contained two C-hordein peptides that were not present in any other LG beer. This class of hordeins is particularly important, as several contain epitopes that are highly immunogenic to CD patients [48]. Figure 2 shows intact epitopes were measured in all beers except for LG1, LG2, and LG9, and the quantitation results measured by targeted MS presented in Figure 3 detected epitopes in all but LG5, LG6, and LG9. All beers except LG9 contained peptides that have been reported to elicit symptoms in CD patients. 

The presence of immunogenic epitopes in LG beers illustrates the technical hurdles to quantifying gluten proteins in fermented foods. Multiple peptides containing the R5 peptide QQPFP were present in LG beers at comparable levels to controls (Figure 3). B-, D-, and γ-hordein peptides were also quantified in LG beers at similar levels to C1, C2, and C3, which gave ELISA gluten estimates over 20 mg/kg. The highest estimate of gluten content in Table 1 is beer C2, with 146 mg/kg gluten, and the LG beers have on average 5.9 mg/kg, some 4% of that of C2. In the net gluten peptide abundance graph, Figure 3, beer C3 has a higher gluten protein content than C2, and the LG beers show a range of values that are approximately 20% that of C2. This gluten content mismatch between techniques may indicate that suppression of ELISA signals is occurring, either by hordein fragments, as hypothesised previously [41], or possibly due to beer polyphenols [57]. 

The Finnish beer LG9 showed remarkably little evidence of gluten protein content. ALP peptide QQQQQGQSFVQPQQQVPVEITR and γ-hordein peptide APFVGVVSGVGGQ were present, as well as low levels of γ- and D-hordeins were detected. The lack of detectable B-hordein peptides suggests they were digested or removed to such a point that they did not cause ELISA signal suppression. The ELISA result of 2.0 mg/kg gluten (Table 1) would therefore be free from suppression by B-hordein fragments, though the ethanol-based extraction solution may mean denatured prolamins were under-estimated [28]. 

These results highlight the difficulties faced when using ELISAs on fermented foods, and the practicality of testing the raw ingredients of fermented foods rather than the fermented or hydrolysed products. Hordein content measurements by R5 ELISA made after fermentation, in the presence of polyphenols, and estimated using certain standard materials have inherent inaccuracies. To ensure that gluten-free-accredited fermented foods contain <10 mg/kg gluten, measurements made using ELISA (or alternatively, LC-MS) must occur prior to fermentation, and the screening of the final product for contaminants must occur using a technique such as LC-MS, which does not encounter the signal suppression issues faced by ELISA. 

## 5. Conclusions

Nine LG beers that were specifically processed to remove gluten had less than 20 mg/kg gluten as measured by ELISA, though in all cases, specific hordein-derived peptides were detected using LC-MS, and in all but one, trace levels of wheat contamination were detected. LC-MS and ELISA measurements of hordein content revealed different relative abundances between the control and LG beers. All nine LG beers tested showed evidence of some hordein content, highlighting the importance of sensitive and reproducible sampling and gluten testing. Three LG beers contained peptides with intact epitopes defined as immunogenic for those with celiac disease, which is concerning because they may elicit symptoms in susceptible individuals if consumed at trace levels. Six LG beers had no immune reactive epitopes, one showed only trace levels of three hordein peptides, and eight of the nine had low levels of wheat gluten peptides, suggesting that contamination occurs at some point in production for most LG beers. This mismatch between ELISA and LC-MS is consistent with previous investigations that demonstrated ELISA-signal inhibition in certain beer types. Investigating the efficacy of gluten-reduction strategies (enzyme use or other proprietary methods) used in various breweries to lower gluten content would require further testing and optimisation. The variation in both total gluten content and presence/absence of immunogenic epitopes across beers that have been processed to remove gluten points to the challenges in gluten-free status assurance. Moreover, gluten presence due to ingredient or equipment contamination poses a further challenge for brewers. These factors are recognised in the recent FDA ruling on the gluten-free labelling of fermented foods. Given the challenges that remain in analysing partially hydrolysed (fermented) products, gluten quantitation of ingredients used in the production of gluten-free beers to test for the presence of contaminants is an appropriate approach, wherein sandwich ELISA is sufficient. Yet for beers produced using gluten-reduction strategies, wherein competitive ELISA may deliver variable results, techniques such as LC-MS offer an alternative for quality control assessment of GR beer production.

## Figures and Tables

**Table 1:** Beers used in analysis and gluten content as measured by R5 competitive ELISA. C = control, LG = low gluten.

Beer Reference Number	Description	Nation of Origin	R5 Competitive ELISA Gluten Concentration (mg/kg)
C1	Adjunct lager	Australia	60.0
C2	Cream ale	Australia	146.3
C3	Strong lager	Denmark	55.9
C4	Adjunct lager	Mexico	9.0
C5	Adjunct lager	Australia	5.4
LG1	Pilsner	Germany	4.7
LG2	Pale lager	Australia	14.7
LG3	IPA	Scotland	4.6
LG4	Pilsner	Scotland	5.3
LG5	Pale lager (Oct 2018)	Finland	4.2
LG6	Pale lager (May 2019)	Finland	4.9
LG7	Pilsner	Finland	7.5
LG8	Helles	Finland	6.0
LG9	Lager	Finland	2.0

## Data Availability

The data presented in this study are available at https://doi.org/10.25919/mfwm-wp25.

## Supplementary Materials

The following supporting information can be downloaded at: https://www.mdpi.com/article/10.3390/foods12173252/s1, Table S1: Protein summary (trypsin results); Table S2: Protein summary (chymotrypsin results).
